# Severe Pulmonary Disease Associated with Electronic-Cigarette–Product Use — Interim Guidance

**DOI:** 10.15585/mmwr.mm6836e2

**Published:** 2019-09-13

**Authors:** Joshua G. Schier, Jonathan G. Meiman, Jennifer Layden, Christina A. Mikosz, Brenna VanFrank, Brian A King, Phillip P. Salvatore, David N. Weissman, Jerry Thomas, Paul C. Melstrom, Grant T. Baldwin, Erin M. Parker, Elizabeth A. Courtney-Long, Vikram P. Krishnasamy, Cassandra M. Pickens, Mary E. Evans, Sharon V. Tsay, Krista M. Powell, Emily A. Kiernan, Kristy L. Marynak, Jennifer Adjemian, Kelly Holton, Brian S. Armour, Lucinda J. England, Peter A. Briss, Debra Houry, Karen A. Hacker, Sarah Reagan-Steiner, Sherif Zaki, Dana Meaney-Delman, CDC 2019 Lung Injury Response Group

**Affiliations:** ^1^National Center for Injury Prevention and Control, CDC; ^2^Wisconsin Department of Health Services; ^3^Illinois Department of Public Health ^4^National Center for Chronic Disease Prevention and Health Promotion, CDC; ^5^Epidemic Intelligence Service, CDC; ^6^National Institute for Occupational Safety and Health, CDC; ^7^National Center for Environmental Health, CDC; ^8^Agency for Toxic Substances and Disease Registry; ^9^Center for Surveillance, Epidemiology and Laboratory Services, CDC; ^10^National Center on Birth Defects and Developmental Disabilities, CDC; ^11^National Center for Emerging and Zoonotic Infectious Diseases, CDC.

*On September 6, 2019, this report was posted as an *MMWR* Early Release on the *MMWR* website (https://www.cdc.gov/mmwr).* As of August 27, 2019, 215 possible cases of severe pulmonary disease associated with the use of electronic cigarette (e-cigarette) products (e.g., devices, liquids, refill pods, and cartridges) had been reported to CDC by 25 state health departments. E-cigarettes are devices that produce an aerosol by heating a liquid containing various chemicals, including nicotine, flavorings, and other additives (e.g., propellants, solvents, and oils). Users inhale the aerosol, including any additives, into their lungs. Aerosols produced by e-cigarettes can contain harmful or potentially harmful substances, including heavy metals such as lead, volatile organic compounds, ultrafine particles, cancer-causing chemicals, or other agents such as chemicals used for cleaning the device (*1*). E-cigarettes also can be used to deliver tetrahydrocannabinol (THC), the principal psychoactive component of cannabis, or other drugs; for example, “dabbing” involves superheating substances that contain high concentrations of THC and other plant compounds (e.g., cannabidiol) with the intent of inhaling the aerosol. E-cigarette users could potentially add other substances to the devices. This report summarizes available information and provides interim case definitions and guidance for reporting possible cases of severe pulmonary disease. The guidance in this report reflects data available as of September 6, 2019; guidance will be updated as additional information becomes available.

Preliminary reports from state health department investigations, a published case series of patients in Illinois and Wisconsin (*2*), and three other published case series (*3*–*5*), describe clinical features of pulmonary illness associated with e-cigarette product use. According to these reports, the onset of respiratory findings, which might include a nonproductive cough, pleuritic chest pain, or shortness of breath, appears to occur over several days to several weeks before hospitalization. Systemic findings might include tachycardia, fever, chills, or fatigue; reported gastrointestinal findings, which have preceded respiratory findings in some cases, have included nausea, vomiting, abdominal pain, and diarrhea. Most identified patients have been hospitalized with hypoxemia, which, in some cases, has progressed to acute or subacute respiratory failure. Patients have required respiratory support therapies ranging from supplemental oxygen to endotracheal intubation and mechanical ventilation. Many patients initially received a diagnosis of infection and were treated empirically with antibiotics without improvement. In the largest cohort, 53 patients from Illinois and Wisconsin (*2*), the six-patient case series in Utah (*4*), and in the five North Carolina patients described in a report in this issue of *MMWR* (*3*), many of the patients who were treated with corticosteroids improved. All patients described in these reports to date have had abnormal radiographic findings, including infiltrates on chest radiograph and ground glass opacities on chest computed tomography scan.

All patients have a reported history of e-cigarette product use, and no consistent evidence of an infectious etiology has been discovered. Therefore, the suspected cause is a chemical exposure. The type, extent, and severity of any chemical-related illness might depend on multiple factors including the chemical to which the user was exposed; chemical changes associated with heating, dose, frequency, and duration of exposure; product delivery methods; and behaviors and medical conditions of the user. The specific behaviors and exposures of identified patients have varied. Most have reported a history of using e-cigarette products containing cannabinoids such as THC, some have reported the use of e-cigarette products containing only nicotine, and others have reported using both. No consistent e-cigarette product, substance, or additive has been identified in all cases, nor has any one product or substance been conclusively linked to pulmonary disease in patients.

Health care providers who cared for the five North Carolina patients diagnosed acute exogenous lipoid pneumonia in all patients based on history of e-cigarette use and clinical, radiographic, laboratory, and bronchoscopy findings. Specifically, the authors identified lipids within alveolar macrophages from the three bronchoalveolar lavage (BAL) specimens stained with oil red O. All five patients reported using marijuana oils or concentrates in e-cigarettes, and three also reported using nicotine (*3*). In a report describing the clinical course and outcomes of six patients from Utah, health care providers described the potential diagnostic utility of identification of lipid-laden macrophages from BAL specimens (*4*). Among the 53 cases from Illinois and Wisconsin, however, the pathologic findings were heterogeneous. Whereas almost half (24/53) of these patients underwent BAL, seven reports described the use of oil red O stain that identified lipid-laden macrophages (*2*). Additional pathologic analyses are in progress on specimens from some of these patients (*2*). The clinical significance of lipid-laden macrophages is currently unclear. It is not known whether the lipid is exogenous (from inhaled material) or endogenous (from altered lipid metabolism). In addition, it is not known whether lipid-laden macrophages are a marker of exposure to e-cigarette aerosol or they are central to the disease process.

CDC is currently coordinating a multistate investigation. Investigations in affected states are focused on describing exposures and the epidemiologic, clinical, laboratory, and behavioral characteristics of cases. In conjunction with a task force from the Council for State and Territorial Epidemiologists and affected states, interim outbreak surveillance case definitions* (Table), data collection tools, and a database to collect relevant patient data have been developed and released. The interim outbreak case definitions will be updated as necessary as additional information becomes available.

**TABLE:** CDC surveillance case definitions* for severe pulmonary disease associated with e-cigarette use — August 30, 2019

Case classification	Criteria
**Confirmed**	Using an e-cigarette (“vaping”) or dabbing^†^ during the 90 days before symptom onset
	**AND**
	Pulmonary infiltrate, such as opacities on plain film chest radiograph or ground-glass opacities on chest computed tomography
	**AND**
	Absence of pulmonary infection on initial work-up: Minimum criteria include negative respiratory viral panel, influenza polymerase chain reaction or rapid test if local epidemiology supports testing. All other clinically indicated respiratory infectious disease testing (e.g., urine antigen for *Streptococcus pneumoniae* and *Legionella*, sputum culture if productive cough, bronchoalveolar lavage culture if done, blood culture, human immunodeficiency virus–related opportunistic respiratory infections if appropriate) must be negative
	**AND**
	No evidence in medical record of alternative plausible diagnoses (e.g., cardiac, rheumatologic, or neoplastic process).
**Probable**	Using an e-cigarette (“vaping”) or dabbing^†^ in 90 days before symptom onset
	**AND**
	Pulmonary infiltrate, such as opacities on plain film chest radiograph or ground-glass opacities on chest computed tomography
	**AND**
	Infection identified via culture or polymerase chain reaction, but clinical team^§^ believes this is not the sole cause of the underlying respiratory disease process **OR** minimum criteria to rule out pulmonary infection not met (testing not performed) and clinical team^§^ believes this is not the sole cause of the underlying respiratory disease process
	**AND**
	No evidence in medical record of alternative plausible diagnoses (e.g., cardiac, rheumatologic, or neoplastic process).

* These surveillance case definitions are meant for surveillance and not clinical diagnosis; they are subject to change and will be updated as additional information becomes available if needed.

^†^ Using an electronic device (e.g., electronic nicotine delivery system (ENDS), electronic cigarette (e-cigarette), vaporizer, vape(s), vape pen, dab pen, or other device) or dabbing to inhale substances (e.g., nicotine, marijuana, tetrahydrocannabinol, tetrahydrocannabinol concentrates, cannabinoids, synthetic cannabinoids, flavorings, or other substances).

^§^ Clinical team caring for the patient.

CDC has provided technical assistance to states and has issued a Clinical Action alert through its Clinician Outreach and Communication Activity network on August 16, 2019 (*6*), and has initiated data collection from states. CDC staff members have deployed to Illinois and Wisconsin, the first states that identified patients, as part of an epidemiologic assistance investigation to assist with their state investigations and continue to work closely with affected states to characterize the exposures and the extent and progression of this illness. CDC is working closely with the Food and Drug Administration (FDA) to facilitate collection of information regarding recent e-cigarette product use among patients and to provide technical assistance related to product samples associated with patients for chemical analysis of remaining substances or chemicals within the e-cigarettes. FDA is focused on processing targeted product samples associated with clinical illness and will analyze samples if there is enough material to test. Those with questions regarding the collection of e-cigarette products for possible testing by FDA should use the following e-mail address: FDAVapingSampleInquiries@fda.hhs.gov.

On August 30, 2019, CDC published recommendations for clinicians, public health officials, and the public based on preliminary information obtained from states and treating clinicians as a Health Advisory (*7*). CDC has created a website (https://www.cdc.gov/tobacco/basic_information/e-cigarettes/severe-lung-disease.html) (*8*) to disseminate up-to-date information and has created a dedicated e-mail address for clinicians and health officials to use to communicate about this public health emergency response (VapingAssocIllness@cdc.gov).

Clinicians are encouraged to consider e-cigarette-associated pulmonary disease as one possible etiology in the broad differential diagnosis of patients with pulmonary disease and a history of e-cigarette product use. Clinicians should evaluate and treat for other possible cases of illness (e.g., infectious, rheumatologic, neoplastic, or other) as clinically indicated. They should report possible cases^†^ to their local or state health department for further investigation.

If e-cigarette product use is suspected as a possible etiology for a patient’s pulmonary disease, a detailed history of the substances used, the sources, and the devices used should be obtained, as outlined in the Health Advisory (*7*), and efforts should be made to determine if any remaining product, devices, or liquids are available for testing. Additional recommendations for clinicians, public health officials, and the public are available and will be updated as needed (*6*–*8*). Clinicians should contact their local or state health departments for further guidance as needed. State public health officials should promptly notify CDC about possible cases and refer to CDC for the most recent versions of the surveillance case definitions, reporting guidelines, and case investigation forms. Public health officials seeking these documents should e-mail CDC at eocevent101@cdc.gov. CDC will revise these tools as new information becomes available and disseminate them to state health departments. General questions regarding this outbreak can be answered by telephoning CDC-INFO (https://www.cdc.gov/cdc-info/index.html).

While this investigation is ongoing and the definitive cause of reported illnesses remains uncertain, persons should consider not using e-cigarette products. Those who do use e-cigarette products should monitor themselves for symptoms (e.g., cough, shortness of breath, chest pain, nausea, vomiting, or other symptoms) and seek medical attention for any health concerns. Regardless of the ongoing investigation, persons who use e-cigarette products should not buy these products off the street and should not modify e-cigarette products or add any substances that are not intended by the manufacturer.

E-cigarette products should never be used by youths, young adults, pregnant women, or by adults who do not currently use tobacco products. Adult smokers who are attempting to quit should use evidence-based smoking cessation treatments, including counseling and FDA-approved medications; those who need help quitting tobacco products, including e-cigarettes, should contact their medical provider. Persons who are concerned about harmful effects from e-cigarette products may call their local poison control center at: 1-800-222-1222. CDC will continue to advise and alert the public as more information becomes available.

**Summary**

**What is already known about this topic?**

Twenty-five states have reported more than 200 possible cases of severe pulmonary disease associated with the use of electronic cigarettes (e-cigarettes).

**What is added by this report?**

Based on available information, the disease is likely caused by an unknown chemical exposure; no single product or substance is conclusively linked to the disease.

**What are the implications for public health practice?**

Until a definitive cause is known, persons should consider not using e-cigarettes. Those who use e-cigarettes should seek medical attention for any health concerns. Clinicians should report possible cases to their local or state health department.

## Footnotes

* Outbreak surveillance case definitions are intended for public health data collection purposes and should not be used as a clinical diagnostic tool or replace individual clinical judgment.

† Clinical illness compatible with the case definition that has not yet been classified.
