# *Erratum:* Vol. 68, No. 36

**DOI:** 10.15585/mmwr.mm6838a4

**Published:** 2019-09-27

In the report “Severe Pulmonary Disease Associated with Electronic-Cigarette–Product Use — Interim Guidance” an author’s affiliation should have read as follows:

Dana Meaney-Delman, MD**^12 12^Division of Birth Defects and Infant Disorders, National Center on Birth Defects and Developmental Disabilities, CDC.**

In addition, the following person should have been included among the members of the CDC 2019 Lung Injury Response Group:

**Livia Navon (Center for Preparedness and Response, Division of State and Local Readiness, assigned to the Illinois Department of Health).**

